# Entropy-Based Combined Metric for Automatic Objective Quality Assessment of Stitched Panoramic Images

**DOI:** 10.3390/e23111525

**Published:** 2021-11-17

**Authors:** Krzysztof Okarma, Wojciech Chlewicki, Mateusz Kopytek, Beata Marciniak, Vladimir Lukin

**Affiliations:** 1 Department of Signal Processing and Multimedia Engineering, West Pomeranian University of Technology in Szczecin, 70-313 Szczecin, Poland; wojciech.chlewicki@zut.edu.pl (W.C.); km46880@zut.edu.pl (M.K.); 2 Faculty of Telecommunications, Computer Science and Electrical Engineering, Bydgoszcz University of Science and Technology, 85-796 Bydgoszcz, Poland; beata.marciniak@pbs.edu.pl; 3 Department of Information and Communication Technologies, National Aerospace University, 61070 Kharkov, Ukraine; lukin@ai.kharkov.com

**Keywords:** image quality assessment, stitched images, panoramic images, image analysis, image entropy

## Abstract

Quality assessment of stitched images is an important element of many virtual reality and remote sensing applications where the panoramic images may be used as a background as well as for navigation purposes. The quality of stitched images may be decreased by several factors, including geometric distortions, ghosting, blurring, and color distortions. Nevertheless, the specificity of such distortions is different than those typical for general-purpose image quality assessment. Therefore, the necessity of the development of new objective image quality metrics for such type of emerging applications becomes obvious. The method proposed in the paper is based on the combination of features used in some recently proposed metrics with the results of the local and global image entropy analysis. The results obtained applying the proposed combined metric have been verified using the ISIQA database, containing 264 stitched images of 26 scenes together with the respective subjective Mean Opinion Scores, leading to a significant increase of its correlation with subjective evaluation results.

## 1. Introduction

Panoramic images, constructed as a result of image stitching operation conducted for a series of constituent images with partially overlapping regions, may suffer from various distortions, including blur, ghosting artifacts, and quite well visible geometric and color distortions. The presence of such issues decreases the perceived image quality and in some cases may be unacceptable from an aesthetic point of view. Although modern cameras and smartphones are usually equipped with software functions making it possible to properly register the overlapping areas of individual photos to create panoramic images, some additional requirements should be fulfilled by users during the recording to prevent such problems. Nevertheless, the growing availability of software and hardware solutions causes higher popularity of panoramic images which may be useful, e.g., as wide background images, in virtual reality scenarios, as well as in mobile robotics for the Visual Simultaneous Localization and Mapping (VSLAM) applications.

Considering the modern applications of image stitching and image registration algorithms, related to the use of cameras mounted on mobile robots, the quality of obtained panoramic images is very important due to potential errors in vision-based control of their motion. In the case of decreased image quality, such images might be removed from the analysis to prevent their influence on the robot’s control. Another interesting direction of such research in mobile robotics concerns the fusion of images acquired by unmanned aerial vehicles (UAVs) [1,2].

One of the most relevant factors, influencing the final quality of the panoramic images, is the appropriate choice of distinctive image features used to match the same regions visible on the “neighboring” constituent images. Nevertheless, some additional post-processing operations conducted after the assignment, such as blending and interpolation, may also have a significant impact on the quality of stitched images. Some obvious examples might be related to different lighting conditions and background changes visible on the constituent images which may cause some easily noticeable seams. Another factor, related to geometric distortions, is the influence of lens imperfections and a too low number of detected keypoints used for further image matching, particularly for constituent images with overlapping areas less than 15–20% of the image area. Although some corrections, e.g., calibration, chromatic aberration or vignetting corrections, may be conducted using both freeware and commercial software for image stitching, even after the final blending some imperfections may still be visible. Since a synchronous acquisition of constituent images using multiple cameras may be troublesome in many practical applications, some problems may also occur for moving objects, particularly leading to motion blur and ghosting artifacts.

Although during several recent years a great progress has been made in general-purpose image quality assessment (IQA), the direct application of those methods proposed by various researches for an automatic objective evaluation of stitched images is troublesome, or even impossible. This situation is caused by significant differences between the most common types of distortions and those which may be found in stitched images. Therefore, the development of stitched images quality assessment methods is limited by the availability of the image databases containing panoramic images subject to different types of distortions typical for image stitching together with subjective quality scores. Since the first attempts to such quality metrics have not been verified using such datasets, there is a need of their additional verification, as well as the analysis of their usefulness in the combination with some other approaches.

Such experiments are possible with the use of the Indian Institute of Science Stitched Image Quality Assessment (ISIQA) dataset consisting of 264 stitched images and 6600 human quality ratings. One of the methods recently proposed for quality assessment of stitched panoramic images, verified using the ISIQA database, is the Stitched Image Quality Evaluator (SIQE) proposed by the authors of this dataset [3]. This method utilized a comparison of 36 features calculated for the constituent and stitched images, namely the eigenvalues determined for the covariance matrix where the covariances are computed for each patch for the pair of wavelet coefficients for a bivariate distribution determined from the Gaussian Mixture Model (GMM) and shape parameters of the Generalized Gaussian Distribution (GGD). The application of the bivariate statistics for the GMM is useful for detection of the correlation caused by ghosting artifacts, whereas the shape parameters of the GGD represent features sensitive to geometric distortions caused by presence of additional edges as well as blur [3]. A more detailed description of the SIQE metric is presented in Section 2.2. Nevertheless, the authors of the SIQE method have used only randomly selected 20% of stitched images for testing whereas 80% of the images have been used for training. Therefore, the reported relatively high correlation results should be considered as harder to obtain for the whole database due to the higher number of images and therefore such overall correlation is significantly decreased [4].

One of the methods for the increase of the correlation of objective metrics with subjective quality evaluation results is the application of the combined metrics, successfully applied for general-purpose IQA [5,6], multiply distorted images, remote sensing [7], as well as for the quality evaluation of the 3D printed surfaces [8]. Although such methods cannot be directly applied for the stitched images, the general idea of the combination of various metrics is worth investigating, leading to promising results as presented in the further sections of the paper.

The motivation for the combination of the entropy-based features with some existing metrics has been related to the observed increase of the local image entropy for the regions containing some kinds of distortions typical for the stitched images. According to expectation, an increase of the global image entropy for lower quality images may also be observed. Nevertheless, as the image entropy is highly dependent on the image contents, a more useful approach is the comparison of the entropy-based features calculated for the constituent and the stitched images in a similar way as for 36 features extracted in the SIQE framework [3]. Hence, the additional entropy-based features may be added after the SVR step and combined with the SIQE values, as well as with some additional features or sub-metrics. The additional use of the variance of the local entropy and two additional features originating from the MIQM metric [9,10], leading to the extension of the idea initially verified in the paper [4], makes it possible to increase the correlations with subjective scores significantly, as presented in the further part of the paper.

## 2. Materials and Methods

### 2.1. Overview of Methods for Stitched Image Quality Assessment

Objective image quality assessment methods may be generally divided into full-reference (FR) and no-reference (NR) methods. The latter group also referred to as “blind” metrics, seems to be more attractive for many applications since FR metrics require the full knowledge of the reference (undistorted) images. Since such “pristine” images are often unavailable, a partial solution for this problem might be the use of the reduced-reference methods where the knowledge of some characteristic parameters or features of the original image is necessary.

Nevertheless, the FR quality assessment of the stitched images should be considered in another way since perfect quality panoramas are usually unavailable, however, there is still a possibility of some comparisons with constituent images that are typically at the disposal. Therefore, the stitched image quality assessment may be considered as an indirect assessment of the quality of the applied stitching method. In view of these assumptions, these methods cannot be directly classified as “purely” FR or NR IQA algorithms, since they use the data from additional (constituent) images but do not utilize any direct comparisons of the distorted panoramas with the “pristine” stitched images.

One of the first interesting approaches to stitched IQA is based on the attempt of using the well-known Structural Similarity (SSIM) method [11] examined by Qureshi et al. [12]. In this method, the SSIM has been used for comparisons of the high-frequency data, e.g., concerning the edges, in overlapping regions of constituent and stitched images, leading to the HFI_SSIM metric. Additionally low-frequency information is used in this metric to assess the photometric quality of the panorama image using the spectral angle mapper (based on the angle between two vectors representing pixels’ colors in the RGB color space) and intensity magnitude ratio measures.

Color correction and balancing in the image and video stitching has also been investigated in the paper [13], whereas the mosaicking performance has been examined by Paalanen et al. [14]. A classification of color correction methods for image stitching can be made using the framework proposed by Bellavia and Colombo [15] who utilize well-known Feature Similarity (FSIM) metric [16] together with the improved Color Image Difference (iCID) measure [17] to assess the quality. Another idea, useful for the analysis of color inconsistency, has been proposed by Niu et al. [18] and is based on the calculation of the color contrast similarity and the color value difference.

The application of the local variance of optical flow field energy between the distorted and reference images has been combined with the intensity and chrominance gradient calculations in highly-structured patches by Cheung et al. [19], allowing mainly for the measurements of the geometric and structure errors.

Unfortunately, regardless of their popularity and good results obtained in some other applications, some data-driven quality assessment methods cannot be successfully applied for the quality assessment of stitched images due to the necessity of training with the use of a great number of images [20]. Some recent examples might be Generated Image Quality Assessment (GIQA) [21] or Face Forensics in the Wild [22] but these methods focus on the evaluation of generated images with distortions different than typical for stitched images or face forgery detection being related to classification rather than quality assessment. Although similar methods might be successfully applied for the general-purpose IQA, it should be kept in mind that for the general-purpose IQA several large-scale databases containing the subjective quality scores are available, differently than for the evaluation of stitched images limited to the use of the ISIQA dataset [3].

One of the methods partially utilized in this paper has been proposed by Solh and AlRegib [9,10] who have developed the Multi-view Image Quality Measure (MIQM) consisting of luminance, contrast, spatial motion, and edge-based structure components. A more detailed description of the application of its simplified version used in our experiments is provided in the further part of the paper (Section 2.3).

### 2.2. The SIQE Metric

As mentioned earlier, one of the most interesting approaches to quality assessment of panoramic stitched images has been recently developed by the inventors of the ISIQA database [3]. The main assumption of the SIQE framework is the use of 36 features divided into two sets. The first set is sensitive to structural changes visible as blurring or changes of edges, whereas the second one captures the distortions caused by variations of the spatial correlation caused by ghosting artifacts. The image-level features are determined as the weighted average of the local patch-level features (calculated for 100×100 pixels patches), further used to predict the final quality score using the Support Vector Machine (SVM) regression.

The detection of ghosting artifacts observed as some additional edges or replications of some image regions, caused by imprecise aligning of the overlapping regions of constituent images during the stitching procedure, is based on the use of the multi-scale multi-orientation decomposition. For this purpose, the use of the steerable pyramids has been proposed by the authors of the paper [3], who have used 2 scales and 6 orientations to decompose the image into 12 subbands. Then, three groups of features may be determined for these subbands both for constituent and stitched images. Fitting a GGD model to subband coefficients their shape coefficients may be determined as the first group of 12 pairs of features. Using the bivariate statistics based on the GMM model, the additional 24 features, representing the covariance values for pairs of wavelet coefficients for the horizontal and vertical neighborhood, may be calculated for the stitched and constituent images, accordingly. These features may be expressed as the eigenvalues of the bivariate distribution [3]. Finally, the differences of all 36 features extracted from the stitched image and the corresponding constituent images (denoted as $f_{1-36}^{s}$ and $f_{1-36}^{c}$) are calculated, being the input for the SVM regression procedure.

Although the Pearson’s Linear Correlation Coefficient (PLCC) for the ISIQA dataset is equal to 0.8395 with Spearman Rank Order Correlation SROCC = 0.8318 reported in [3], these results have been obtained for 1000 iterations of randomly chosen train and test sets, using only 20% of images for testing. Unfortunately, applying this metric for the whole ISIQA dataset, significantly lower values of the PLCC = 0.7488 and SROCC = 0.7057 may be achieved [4].

Considering the necessity of the use of a large amount of the ground truth data for training to avoid overfitting of the trained model, there is a limited possibility of application of the deep CNN-based methods, as stated by Hou et al. [20]. For this reason, regardless of the popularity of the deep learning methods, an interesting direction of research seems to be the development of combined metrics, utilizing the SIQE method and some other approaches based on handcrafted features.

### 2.3. The Simplified MIQM Implementation

Although the fundamental element for our research is the SIQE metric, its extension towards a combined metric requires an implementation of some additional metrics and calculation of additional features, as well as their further optimization making it possible to increase the correlation with subjective quality scores.

Two additional sub-measures have been incorporated from [9] for this study, i.e., luminance and contrast index, and edge-based structural index, being the elements of the MIQM. The first one is focused on recognizing sharp local changes in luminance and contrast around structured regions. The computation formula was derived and adjusted in [10] to provide higher weights for structured regions. It is mathematically expressed in the following way

(1) $$k_{x,y}\left(I,J\right)=\frac{4\left(\sigma_{I}\sigma_{J}\right)\cdot \left(\mu_{I}\mu_{J}\right)+C}{\left(\sigma_{I}^{2}+\sigma_{J}^{2}\right)\cdot \left(\mu_{I}^{2}+\mu_{J}^{2}\right)+C}$$

where (x,y) is the coordinate of the upper left corner of the macroblock. The mean intensity is denoted as μ, and the standard deviation as σ, respectively. Both σ and μ are computed for the macroblocks of the dimension s×s. In our study, we have set *s* to 21, which is a trade-off between a reasonable computation time and accuracy. The subscript *I* denotes the reference image whereas *J* stands for the distorted image, and *C* is a constant added to prevent instability in case of the denominator value being close to zero.

To compute the overall quality index a weighted average of luminance and contrast index of each macroblock should be used. The weights’ values are obtained based on the reference image in the following way. First, the texture randomness index at macroblock (x,y) of the image *I* has to be computed using the formula [23]

(2) $$t\left(x,y\right)=E_{I}\left(x,y\right)\times M_{I}\left(x,y\right)$$

where $E_{I}$ is an edge intensity binary image with values equal to 1 where the function recognizes edges, and values 0 elsewhere, with $M_{I}$ being the mean intensity of *I*. These values have been computed for the same non-overlapping macroblocks as previously. Finally, the texture randomness index has been mapped to the object index in the following way

(3) $$T\left(x,y\right)=\left\{\begin{array}{cc} K_{1}+\left(0.5\times K_{1}\times \frac{\log_{2}t\left(x,y\right)}{\log_{2}\beta_{1}}\right) & \beta_{1}\leq t\left(x,y\right)<\beta_{2}\\ K_{2}+\left(0.5\times K_{2}\times 2^{-(t\left(x,y\right)-\beta_{2})}\right) & t\left(x,y\right)\geq \beta_{2}\\ K_{1} & \mathrm{otherwise} \end{array}\right.$$

where $K_{1}$ and $K_{2}$ are the constant parameters that control the weights assigned to the structured regions and randomly assigned regions, accordingly. If $K_{1}$ is much larger than $K_{2}$, then higher weights are assigned to the structured regions. Parameters $\beta_{1}$ and $\beta_{2}$ are the edge detector thresholds. Such computed T(x,y) is employed to compute both sub-measures according to the Formulas (4) and (5).

The luminance and contrast index for M×N macroblocks may be calculated as

(4) $$K\left(I,J\right)=\frac{\sum_{x=1}^{M}\sum_{y=1}^{N}k_{x,y}\left(I,J\right)\times T\left(x,y\right)}{\sum_{x=1}^{M}\sum_{y=1}^{N}T\left(x,y\right)}$$

whereas the edge-based structural index for M×N macroblocks is defined as

(5) $$E\left(I,J\right)=\frac{\sum_{x=1}^{M}\sum_{y=1}^{N}\left(1-∥\frac{T_{x,y}\left(I\right)-T_{x,y}\left(J\right))}{T_{x,y}\left(I\right)}∥\right)}{M\times N}\;.$$

The values of K(I,J) and E(I,J) are close to 1 for minimum distortions and consequently values almost 0 for maximum distortions.

In our study, the reference image has been a region of interest (ROI) selected from each constituent image and the corresponding ROI found in the evaluated stitched image. All the formulas have been implemented as MATLAB functions. Instead of the third MIQM term, namely spatial motion index, partially utilizing the local entropy, we have used the additional global and local entropy-based features, leading to the increase of the proposed metric’s correlation with subjective MOS values.

### 2.4. The Proposed Entropy-Based Approach

**Figure 1:**
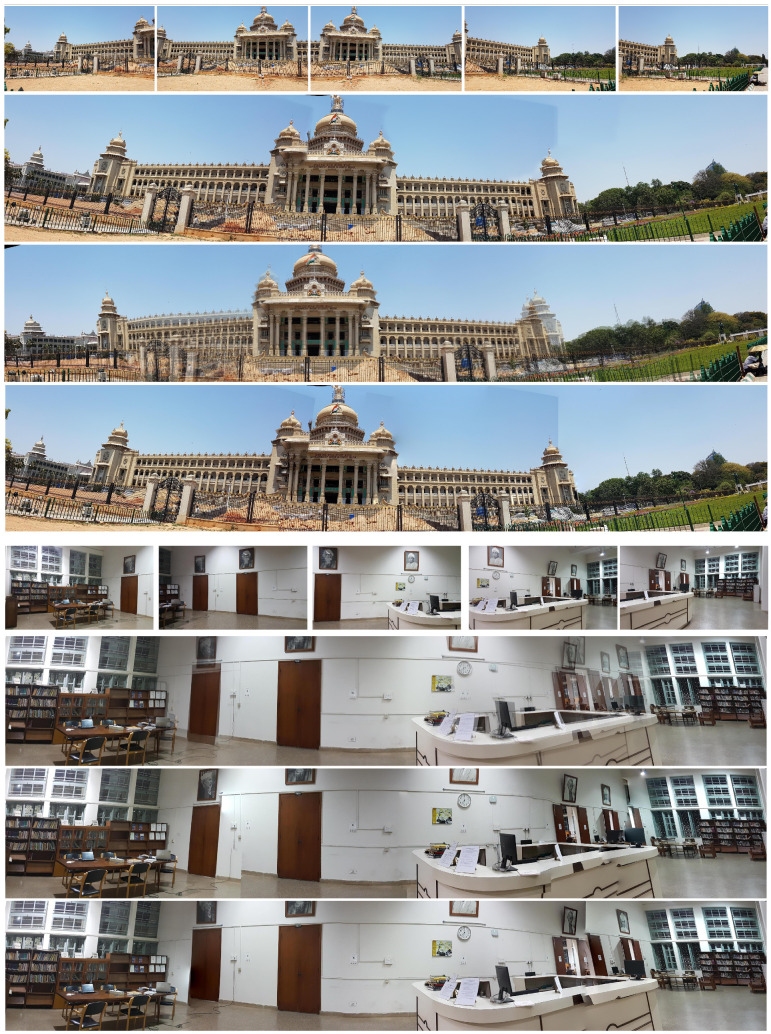
Sample constituent and stitched images with various distortions from the ISIQA dataset.

The initial experiments, conducted using 264 stitched images obtained for 26 scenes that are included in the ISIQA dataset (sample images are shown in Figure 1) as well as some additional stitched images generated using the freeware Hugin software with various parameters, have demonstrated the potential improvements of existing metrics caused by their diversity.

Assuming the usefulness of the image entropy, reflecting the amount of information in an image, the first experiments have been made utilizing the global entropy values calculated for the image *X* according to the well-known formula:

(6) $${ent}_{global}^{X}=-\sum \left(p\cdot \log_{2}\left(p\right)\right)\;,$$

where *p* contains the normalized histogram counts determined for the image *X* using 256 bins, as well as the local entropy values calculated using the same Formula (6) for the 9×9 pixels neighborhood of the specified pixel from the image *X*.

Therefore, the additional entropy-based features (added after the SVR step and further combined with the SIQE values) are defined as [4]:

(7) $${\bar{ent}}_{local}={\bar{ent^{c}}}_{local}-{\bar{ent^{s}}}_{local}\;,$$

and

(8) $$ent_{global}=ent_{global}^{c}-ent_{global}^{s}\;,$$

where the average local entropy values ${\bar{ent}}_{local}$ and the global entropy values $ent_{global}$ for constituent (*c*) and stitched (*s*) images are subtracted, respectively. Regardless of these two differential features, their equivalents for the constituent and stitched images may be independently analyzed as well.

After the experimental verification of the possible combinations, the initially considered combined metrics, referred to as EntSIQE, have been defined in two variants based on the weighted sum and weighted product [4]:

(9) $${\mathrm{EntSIQE}}_{1}=\alpha \cdot \mathrm{SIQE}+\beta \cdot ent_{global}+\gamma \cdot {\bar{ent^{s}}}_{local}\;,$$

and

(10) $${\mathrm{EntSIQE}}_{2}={\left(\mathrm{SIQE}\right)}^{\alpha }\cdot {\left(ent_{global}\right)}^{\beta }\cdot {\left({\bar{ent^{s}}}_{local}\right)}^{\gamma }\;,$$

where the parameters α, β, and γ may be optimized (independently for each of the above formulas) to provide the highest correlation with the MOS values for the specified database, e.g., the ISIQA database as used in this paper. Since the use of the averaged ${\bar{ent}}_{local}$ features, calculated only for the stitched images, has provided better results than the use of the differences for the constituent and stitched images, only the $ent_{global}$ features have been calculated as the difference of features for the constituent and stitched images.

The additional extension of these metrics with the use of two indexes, originating from the MIQM approach (Equations (4) and (5)), described in Section 2.3, may be conducted in the same way, leading to the finally proposed metrics referred to as ${\mathrm{EntSIQE}}_{1}^{+}$ and ${\mathrm{EntSIQE}}_{2}^{+}$. Nevertheless, applying the Formulas (4) and (5), two vectors containing the similarity indexes between the ROIs extracted from the stitched image and each corresponding constituent image may be obtained. Therefore, for the images from the ISIQA database, depending on the number of constituent images (four in two sets and five in the other cases), 8 or 10 values may be obtained in aggregate. Considering the use of the maximum, minimum, average and median values for K(I,J) and E(I,J) indexes, the best results have been obtained using the median values.

The additional useful feature, leading to a further increase of the correlation of the proposed metric with subjective scores is the variance of the local entropy that may be calculated subtracting the averaged variances determined for the constituent and stitched images according to:

(11) $${\bar{var}}_{ent}=\bar{var}\left({ent^{c}}_{local}\right)-\bar{var}\left({ent^{s}}_{local}\right)\;.$$

Hence, the final formulas may be expressed as:

(12) $$\begin{array}{c} {\mathrm{EntSIQE}}_{1}^{+}=a_{1}\cdot {\left(\mathrm{SIQE}\right)}^{w_{1}}+a_{2}\cdot {\left(ent_{global}\right)}^{w_{2}}+a_{3}\cdot {\left({\bar{ent^{s}}}_{local}\right)}^{w_{3}}\\ +a_{4}\cdot {\left({\bar{var}}_{ent}\right)}^{w_{4}}+a_{5}\cdot {\left(med\left(K(I,J)\right)\right)}^{w_{5}}+a_{6}\cdot {\left(med\left(E(I,J)\right)\right)}^{w_{6}} \end{array}\;\;,$$

and

(13) $$\begin{array}{c} {\mathrm{EntSIQE}}_{2}^{+}={\left(\mathrm{SIQE}\right)}^{w_{1}}\cdot {\left(ent_{global}\right)}^{w_{2}}\cdot {\left({\bar{ent^{s}}}_{local}\right)}^{w_{3}}\\ \cdot {\left({\bar{var}}_{ent}\right)}^{w_{4}}\cdot {\left(med(K(I,J))\right)}^{w_{5}}\cdot {\left(med(E(I,J))\right)}^{w_{6}} \end{array}\;\;.$$

It is worth to note that the necessity of the use of additional weighting exponents in Formula (12) in comparison to the Formula (9) is caused by different dynamic ranges of individual features used in the proposed metrics. Nevertheless, the application based on the weighted product might be a better choice due to the use of only six weighting coefficients ($w_{1}$–$w_{6}$).

## 3. Results and Discussion

To verify the correlation between the objective and subjective quality scores for the 264 images from the ISIQA database, three correlation metrics being the most typical in the IQA research, have been used.

Pearson’s Linear Correlation Coefficient (PLCC) between the objective metric Q the Mean Opinion Score (MOS) values, illustrating the prediction accuracy of the image quality, is defined as the ratio of the covariance to the product of the standard deviations:

(14) $$r=\frac{\mathrm{cov}(Q,MOS)}{\sigma_{Q}\cdot \sigma_{MOS}}.$$

It should be noted that in many IQA related papers, the additional nonlinear regression is applied, usually with the use of the logistic function, according to the recommendations of the Video Quality Experts Group (VQEG). Nevertheless, in the case of the combined metrics, it does not lead to meaningful changes of the correlation coefficients (differences are typically below 0.003) due to the nonlinear combination of various features. This has also been verified experimentally both for the original SIQE metric as well as for all the proposed combinations.

To verify the prediction monotonicity, two rank-order correlations may be applied. Spearman’s Rank Order Correlation Coefficient (SROCC) is given as:

(15) $$\rho =1-\frac{6\cdot \sum d_{i}^{2}}{n\cdot (n^{2}-1)},$$

where $d_{i}$ stands for the difference between the ranks of corresponding images in two sets sorted according to objective (Q) and subjective (MOS) quality scores and *n* denotes the number of images. The second one is Kendall Rank Order Correlation Coefficient (KROCC) expressed as:

(16) $$\tau =2\cdot \frac{n_{c}-n_{d}}{n\cdot (n-1)},$$

where $n_{c}$ and $n_{d}$ are the number of concordant and discordant, respectively, that are considered as the pairs of images ordered in the same way and reversely.

The calculations of all parameters as well as the optimizations have been conducted in MATLAB environment. For the optimization of exponential parameters $w_{i}$ as well as the multipliers $a_{i}$ the derivative-free method without constraints based on the Nelder–Mead simplex method has been used in the version implemented in MATLAB’s *fminsearch* function with additional verification of the local minima.

**Figure 2:**
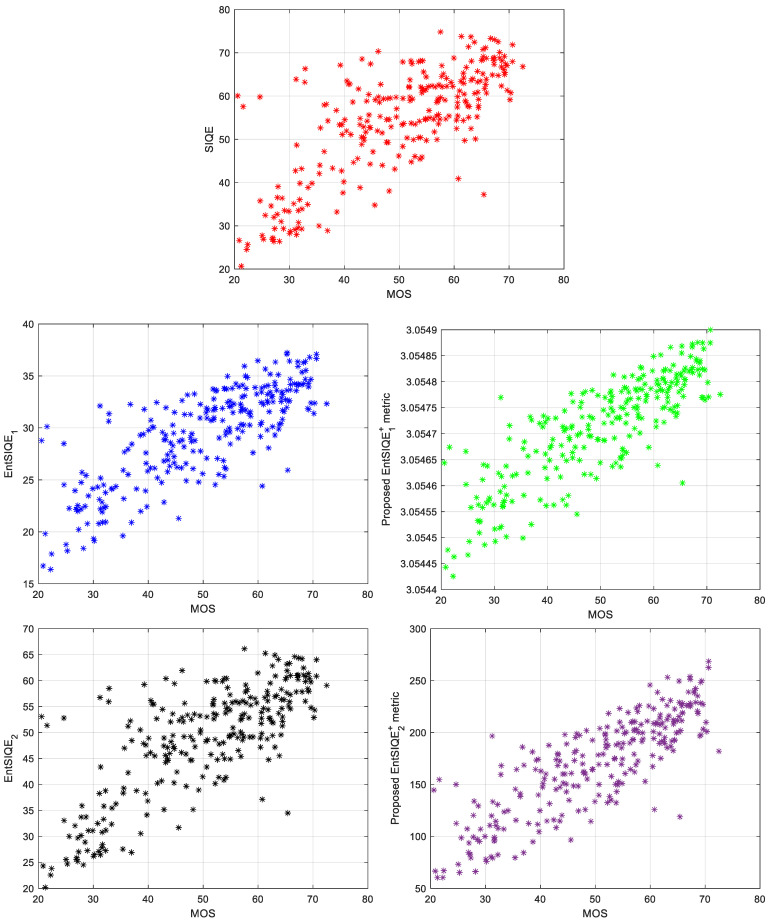
Scatter plots for SIQE (red points), two variants of the EntSIQE metric (blue and black points), and the proposed two variants of the EntSIQE${}^{+}$ metric (green and violet points).

The obtained results for the original SIQE metric as well as for the initially considered and finally proposed combined metrics are presented in Table 1 and Table 2 as well as on the scatter plots shown in Figure 2. Since some comparisons of the original SIQE with the older metrics (i.a. NIQE, BRISQUE, DIVIINE or HFI_SSIM [12]), presented in the paper [3], have demonstrated its significantly better performance with correlation’s increase by over 0.2 for the same ISIQA dataset, the analysis in this paper is limited to the comparison to the state-of-the-art SIQE metric to increase the clarity of presented results.

As can be easily noticed much more linear relation between the proposed objective metrics and MOS values can be observed in comparison to the original SIQE metric. Analyzing the number and location of outliers, most of them are located closer to the linear trend visible on the scatter plots for the proposed metrics. The values of the parameters obtained for the proposed combined metrics are presented in Table 3.

Analyzing the obtained results, a significant increase of the correlation with subjective scores may be observed for the proposed approach. Since the values of the parameters used for all six metrics or features are not close to zero (for the EntSIQE${}_{1}^{+}$ metric there is no pair of parameters $a_{i}$ and $w_{i}$ being close to zero), a removal of any of the parts of the combined metric would decrease the correlation of the combined metric with the MOS values. To illustrate this, the results of the ablation study (with independent optimization) are presented in Table 2 for the six versions of the 5-element combined metrics (with removed one of the components). As it may be easily noticed, in both cases the most relevant element is undoubtedly the original SIQE metric. Nevertheless, only a slightly lower correlation with subjective scores may be achieved without the use of the global entropy or median values of (K(I,J)) calculated according to Formula (4).

## 4. Conclusions

The extensions of the recently proposed SIQE metric towards the combined metric proposed in the paper make it possible to achieve considerably higher correlation of the designed objective metrics with subjective quality scores of the stitched images delivered in the ISIQA database. The obtained results are promising and confirm the usefulness of the combined metrics also for the automatic quality assessment of the stitched panoramic images. As shown in the ablation study, the application of the additional entropy-based features utilizing the local image entropy and its variance is one of the crucial elements increasing the correlation with the MOS values, since their removal causes the most significant decrease of the PLCC, SROCC and KROCC values, obviously with the exception of the original SIQE metric.

One of the potential directions of further research might be the application of the proposed approach for quality assessment of parallax-tolerant image stitching methods [24] as well as the validation of the proposed approach for some other types of images and video sequences, containing similar types of distortions, also using some other combination models.

## Figures and Tables

**Table 1:** Correlations with subjective scores obtained for the ISIQA database for the initially considered metrics and their parameters [4].

Metric	Parameters			Correlation with MOS		
	α	β	γ	PLCC	SROCC	KROCC
SIQE	-	-	-	0.7488	0.7057	0.5308
EntSIQE${}_{1}$	0.3592	0.7938	2.3176	0.8012	0.7920	0.5971
EntSIQE${}_{2}$	0.8597	−0.0036	0.4579	0.8101	0.7945	0.5990

**Table 2:** Correlations with subjective scores obtained for the ISIQA database using the proposed combined metrics and during the ablation study assuming the removal of one of the elementary metrics or features.

Metric	Correlation with MOS		
	PLCC	SROCC	KROCC
EntSIQE${}_{1}^{+}$ (proposed)	0.8338	0.8338	0.6418
EntSIQE${}_{1}^{+}$ with $a_{1}=0$	0.2869	0.2859	0.1833
EntSIQE${}_{1}^{+}$ with $a_{2}=0$	0.8319	0.8326	0.6401
EntSIQE${}_{1}^{+}$ with $a_{3}=0$	0.8283	0.8267	0.6341
EntSIQE${}_{1}^{+}$ with $a_{4}=0$	0.8288	0.8264	0.6334
EntSIQE${}_{1}^{+}$ with $a_{5}=0$	0.8326	0.8335	0.6417
EntSIQE${}_{1}^{+}$ with $a_{6}=0$	0.8295	0.8250	0.6312
EntSIQE${}_{2}^{+}$ (proposed)	0.8337	0.8341	0.6432
EntSIQE${}_{2}^{+}$ with $w_{1}=0$	0.2652	0.2870	0.1885
EntSIQE${}_{2}^{+}$ with $w_{2}=0$	0.8336	0.8336	0.6424
EntSIQE${}_{2}^{+}$ with $w_{3}=0$	0.8128	0.8037	0.6130
EntSIQE${}_{2}^{+}$ with $w_{4}=0$	0.8282	0.8225	0.6296
EntSIQE${}_{2}^{+}$ with $w_{5}=0$	0.8333	0.8311	0.6399
EntSIQE${}_{2}^{+}$ with $w_{6}=0$	0.8273	0.8249	0.6309

**Table 3:** Parameters obtained for the ISIQA database for the newly proposed metrics.

Metric	Parameters					
	$w_{1}$	$w_{2}$	$w_{3}$	$w_{4}$	$w_{5}$	$w_{6}$
EntSIQE${}_{1}^{+}$	0.9010	1.8909	$1.534\times 10^{-4}$	1.4566	$9.359\times 10^{-5}$	$2.438\times 10^{-4}$
EntSIQE${}_{2}^{+}$	1.1574	−0.0037	0.5554	0.0262	0.1271	1.1078
	$a_{1}$	$a_{2}$	$a_{3}$	$a_{4}$	$a_{5}$	$a_{6}$
EntSIQE${}_{1}^{+}$	$1.391\times 10^{-5}$	$1.582\times 10^{-4}$	3.1237	$2.079\times 10^{-4}$	0.9362	1.6689

## Data Availability

Not applicable.

## Abbreviations

The following abbreviations are used in this manuscript:

CNN	Convolutional Neural Network
FR IQA	Full-Reference Image Quality Assessment
FSIM	Feature Similarity
GIQA	Generated Image Quality Assessment
GGD	Generalized Gaussian Distribution
GMM	Gaussian Mixture Model
iCID	improved Color Image Difference
ISIQA	Indian Institute of Science Stitched Image Quality Assessment (dataset)
KROCC	Kendall Rank Order Correlation Coefficient
MIQM	Multi-view Image Quality Measure
MOS	Mean Opinion Scores
NR IQA	No-Reference Image Quality Assessment
PLCC	Pearson’s Linear Correlation Coefficient
ROI	region of interest
SIQE	Stitched Image Quality Evaluation
SROCC	Spearman Rank Order Correlation Coefficient
SVM	Support Vector Machine
UAV	Unmanned Aerial Vehicle
VQEG	Video Quality Experts Group
VSLAM	Visual Simultaneous Localization and Mapping
